# Fecal microbiota transplants: A review of emerging clinical data on applications, efficacy, and risks (2015–2020)

**DOI:** 10.5339/qmj.2021.5

**Published:** 2021-02-22

**Authors:** Dana Al-Ali, Aamena Ahmed, Ameena Shafiq, Clare McVeigh, Ali Chaari, Dalia Zakaria, Ghizlane Bendriss

**Affiliations:** ^1^Premedical Division Weill Cornell Medicine-Qatar, Premedical Division, PO Box 24144 Doha, Qatar E-mail: ghb2002@qatar-med.cornell.edu; ^2^Northwestern University Qatar, Doha, Qatar

**Keywords:** Fecal transplant, microbiota, clinical trial, antibiotic resistance, *Clostridium difficile*, Qatar

## Abstract

As the importance of the gut microbiota in health and disease is a subject of growing interest, fecal microbiota transplantation (FMT) was suggested as an attractive therapeutic strategy to restore homeostasis of the gut microbiota, thereby treating diseases that were associated with alteration of the gut microbiota. FMT involves the administration of fresh, frozen, or dried fecal microorganisms from the gut of a healthy donor into the intestinal tract of a patient. This rediscovery of the potential benefits of an ancient practice was accompanied by a rapid progression of our understanding of the roles and mechanisms of gut microbes in the pathogenesis of disease. With a growing number of diseases being associated with dysbiosis or the alteration of gut microbiota, FMT was suggested as an attractive therapeutic strategy to “reset the gut” and initiate clinical resolutions or remissions. The number of FMT clinical trials is increasing worldwide, but no trials are registered in the Gulf region; this suggested the need for raising awareness of the latest studies on FMT. This review presented the emergent preclinical and clinical data to give an overview of the potential clinical applications, the benefits, and inconveniences that were worth considering for eventual future testing of fecal transplants in Qatar and the Middle East. This study highlighted the diversity of methods tested and commented on the variables that can affect the assessment of the effectiveness of FMT in specific diseases. The risks associated with FMT and the threat of antimicrobial resistance for this therapeutic approach were reviewed. From gastrointestinal diseases to neurodevelopmental disorders, understanding the roles of the gut microbiota in health and disease should be at the heart of developing novel, standardized, yet personalized, methods for this ancient therapeutic approach.

## Introduction

**Figure 1.**
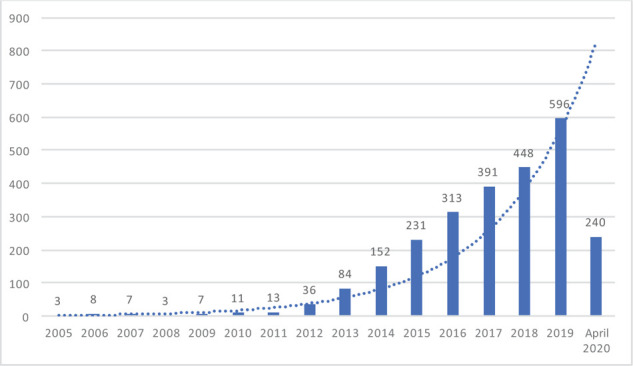
**Growing scientific interest toward fecal transplants over the past 15 years.** A search using the MeSH term “fecal transplant” was done on PubMed and filtered for the last 15 years.

Over the past few decades, the importance of the human gut microbiome and its central role in revolutionizing the way the disease is understood and treated has become increasingly evident. As several diseases and disorders have been associated specifically with gut dysbiosis,^1–7^ a new therapeutic approach that aims at instantly correcting the gut dysbiosis emerged: the fecal microbiota transplantation (FMT). This therapy is currently the focus of heated discussions between scientists, clinicians, and the general public^8^ (Figure 1).

**Figure 2.**
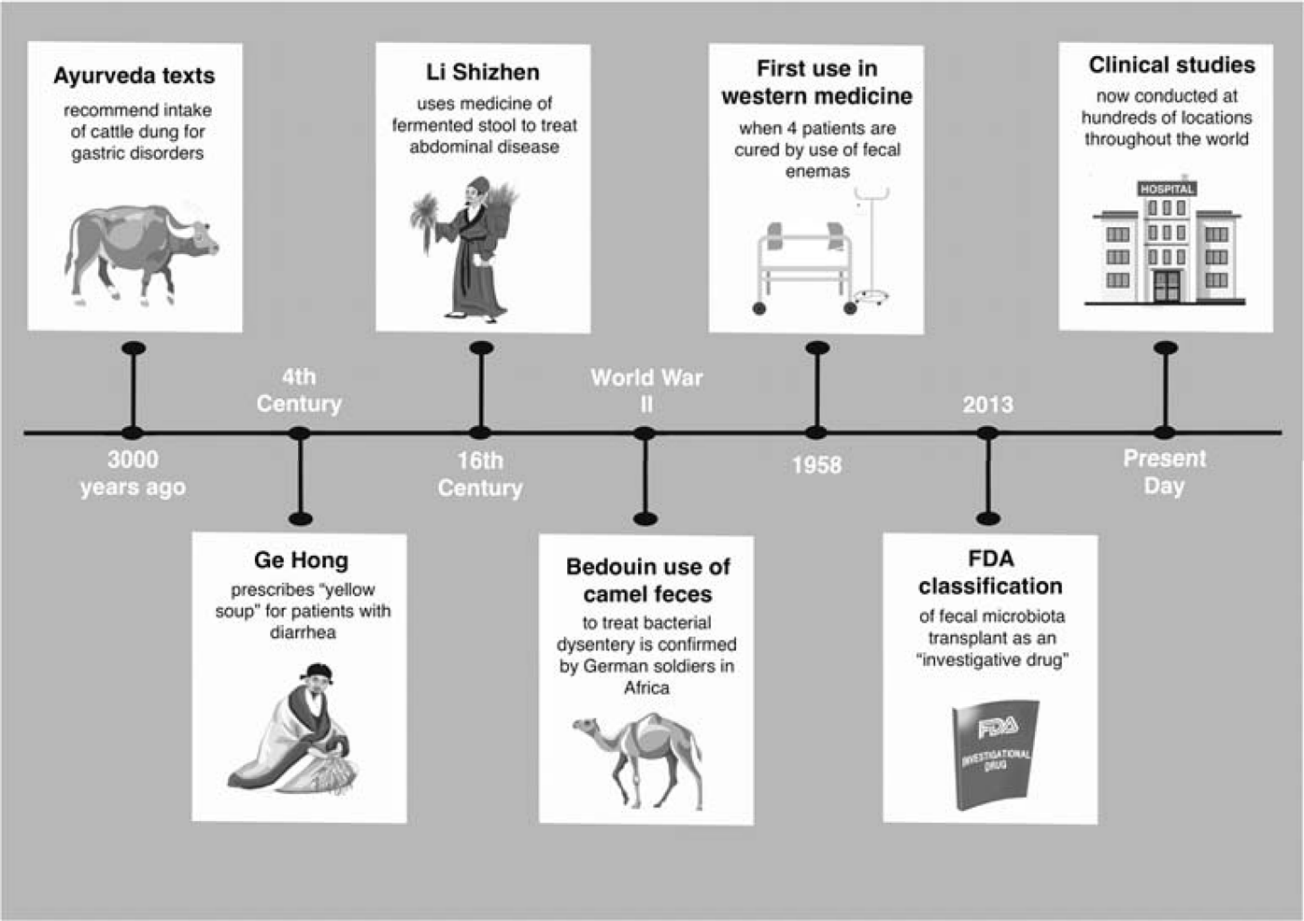
Key milestones in the history of fecal transplants.

FMT involves the transfer of microbiota in the form of feces from a human stool donor to the gastrointestinal tract of another patient. It is typically administered through enemas, colonoscopy, or to the upper gastrointestinal tract with a nasal tube. More recent applications include using frozen or freeze-dried/lyophilized material in place of fresh stool.^9^ These methods have mixed benefits, risks, and inconvenience (Figure 2).

Allocoprophagy is the scientific term for ingesting the feces of another individual. This practice has been observed in animals both in captivity and the wild. Cases involving elephants, mice, mole rats, pigs, and chimpanzees have all been well documented.^10^ The majority of cases appear to involve the ingestion of maternal feces by their young. The speculation that this is a strategy to colonize the sterile digestive tracts of neonates with helpful bacteria has prompted several scientific studies. For example, it was observed that piglets who ingested maternal feces during the weaning period showed improved immunocompetence and faster growth.^11^ Similarly, wild mice have been found to reinoculate their microbiome with bacteria to maintain levels of *Lactobacillus*. This has been recreated in a laboratory setting where mice practicing allocoprophagia showed a higher volume and diversity of their gut microbiome when compared to the control group.^12^

Interspecies allocopraphagy has been practiced in human societies for thousands of years. The earliest evidence comes from India 3000 years ago in the form of ancient Ayurveda texts advocating the intake of cattle dung for gastric disorders.^13^ However, the earliest record of allocopraphagy involving human feces does not occur until the 4th century when the Chinese physician Ge Hong prescribed the “yellow soup” for patients suffering from diarrhea. Similar practices persisted in traditional Chinese medicine for hundreds of years as illustrated by the 16th century writings of Li Shizhen.^14^ In fact, fecal suspensions involving both human and nonhuman material seem to have been used historically by a variety of cultures as evidenced by several reports retrieved from World War II. For example, German soldiers reported the Bedouin practice of using camel dung to treat bacterial dysentery.^15^ The use of FMT did not occur in western medicine until 1958 when a team of American surgeons used fecal transplants to successfully treat *Clostridium difficile* (CDI) infections (fulminant pseudomembranous colitis) in four critically ill patients.^16^

Because the efficacy of FMT in treating *C.* *difficile* infections started to become evident and the use of FMT spread widely,^17^ the US Food and Drug Administration classified FMT as an investigational drug in July 2013; this resulted in significant limitations of its use by subjecting it too expensive and stringent criteria.^18^ This step has been met with much criticism from both doctors and patients.^19^ Meta-analyses have shown that FMT is indeed an effective treatment for recurrent and refractory C. *difficile* infections.^20^

Nevertheless, there is growing evidence of the therapeutic potential of targeting the gut microbiome for treatment of other diseases beyond C. *difficile* infections, such as inflammatory bowel diseases, diabetes, obesity, metabolic syndrome, cardiovascular diseases, various types of cancers, and neurodevelopmental disorders such as autism.^21,22^ Preclinical studies using animal models have shown how diabetes and obesity can be transferred from humans to germ-free mice via a fecal transplant,^23^ and how diseases such as diabetes, obesity, or even behavioral disorders can be reversed in mice using FMT.^24–28^ These preclinical studies have encouraged the start of hundreds of clinical trials using FMT to treat most types of diseases and disorders, including cancers, neurological diseases, neuropsychiatric disorders, gastrointestinal diseases, liver and renal diseases, metabolic syndrome, and hypertension.^29^

This review provides a brief update on recently published and ongoing clinical trials worldwide as evidence supporting the use of FMT beyond the case of CDI is lacking. It also presents a discussion of the potential clinical applications and the benefits and risks that need to be considered for eventual future testing of fecal transplants in Qatar and the Middle East. A study by Al Thani and colleagues^30^ (2014) has reported the prevalence of CDI in Qatar to be 7.9%, which is higher than Europe. The study also identified strains that are different from the ones found in America, Europe, and Asia. These findings highlight the importance of local surveillance to detect and control CDI. Although prevalence of inflammatory bowel disease (IBD) in Qatar has not been clearly established, some studies show that the frequency of IBD is relatively high and includes atypical cases.^31–33^

As more than half of the trials are testing FMT on C. *difficile* and gastrointestinal diseases, this study reviewed those studies and their results. In order to raise awareness in Qatar on the extradigestive clinical applications of FMT, this study will reveal the results of FMT trials on diabetes, obesity, and autism spectrum disorders (ASDs), which have been shown to be of higher prevalence in Qatar than the worldwide averages.^34–36^

## Methods

The database *clinicaltrials.gov* was accessed on April 23rd, 2020, using the expression “fecal transplant” under the condition or disease search window. The count of “active or completed trials” was obtained using the filter to exclude all trials that were labeled as withdrawn, of unknown status, terminated, or suspended. Descriptive statistics were performed on what we have labeled as “active or completed” trials using Microsoft Excel to generate the pie charts. Results of clinical studies for selected diseases were compiled and compared in tables. Selected diseases include *C.* *difficile* infections, IBD, diabetes, obesity, and ASDs.

Another search was performed on PubMed database using the MeSH terms “fecal transplant” in all fields with a filter for the past five years (2015–2020). All types of studies were included in the search. Publications were scanned for relevant information facilitating discussion of applications, efficacy, and risks in preclinical and clinical studies.

## Clinical Trials

### Clinical trials worldwide

**Figure 3.**
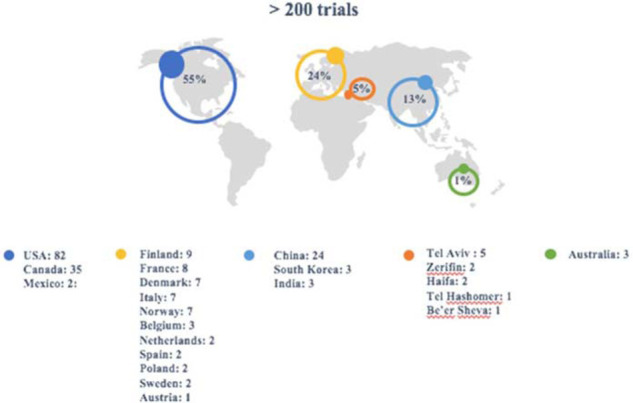
**Worldwide distribution of fecal transplants clinical trials as of May 19, 2020.** A total of 215 trials registered in database *clinicaltrials.gov* with search term “fecal transplant.” Trials that were labeled as withdrawn, terminated, or suspended were excluded.

More than 200 clinical trials testing fecal transplants were active or completed and registered on the *clinicaltrials.gov* database that have met the criteria mentioned in the methods section (were not labeled as withdrawn, unknown status, terminated, and suspended) (Figure 3). Another 30 studies were either withdrawn, terminated, or suspended, and 40 studies had an unknown status for their recruitment. Reasons for withdrawal, suspension, or termination can be varied and might include no participants or very low number of participants, serious adverse events, or funding issues. The 30 trials that were withdrawn, suspended, or terminated were in Europe (3 trials), Israel (4 trials), China (1 trial), Canada (5 trials), and USA (13 trials).

This review focuses on the active or completed trials. Most of these trials are performed in the United States, China, Canada, and Europe (Figure 3). Fecal transplant clinical trials on CDI account for 75% of *infectious disease* and 23.6% of *all* fecal transplant clinical trials worldwide. This means that more than 70% of trials using FMT are being performed to investigate diseases other than CDI.

**Figure 4.**
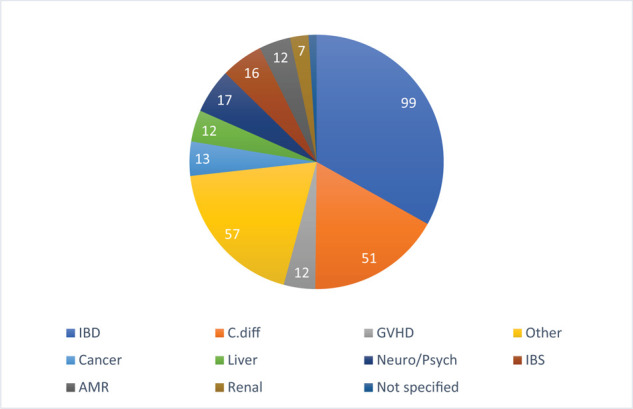
**Worldfecal transplants clinical trials (number per disease category)**. Abbreviations: IBD = inflammatory bowel disease; AMR = antibiotic resistance; GVDH = graft versus host disease; C. diff = Clostridium difficile infection; Neuro/psych = neurological diseases and neuropsychiatric disorders; IBS = irritable bowel syndrome. Data were obtained from clinicaltrials.gov as per method detailed in section 2.

FMT is being tested for various diseases including IBD with 25.9% of all trials, irritable bowel syndrome (IBS), other gastrointestinal disorders (GI), CDI, graft versus host disease (GVDH), cancer, liver disease, neurological diseases and neuropsychiatric disorders, antimicrobial resistance, endocrine diseases (endo), rheumatology, renal diseases, and other nonspecified diseases (Figure 4). Studies that have published their results are mostly for CDI and IBD and are listed in Table 1 and Table 2. Until recently, no trials have been registered in the Middle East. However, one trial testing the efficacy of FMT for IBD has been registered at Gulhane Medical Academy, Ankara, Turkey; the recruitment status of this study is unknown.

#### C. difficile infections

*C. difficile* is a gram-positive, anaerobic, spore-forming, toxin-producing bacillus, which was renamed in 2016 as *Clostridioides difficile.* One of the first applications of the last century was in 1958 for the treatment of recurrent CDI.^5^ The infection causes the release of toxin in amounts large enough to damage the intestinal barrier resulting in poor ion absorption by the body and consequent severe inflammation of the colon. *C*. *difficile* is the leading cause of healthcare-associated infections worldwide.^37^ Given the widespread use of antibiotics, approximately 453,000 incidences of CDI cases were observed in the United States in 2011 alone, many of which were fatal.^38^ Dr. Ben Eiseman, an American surgeon, used fecal enemas to treat the symptoms of severe diarrhea in an attempt to restore healthy gut microbiota.^39^ This method was used as an alternative to other forms of treatment, such as antibiotics, which are known to alter the structure of the human gut creating further dysbiosis and thus yielding unsuccessful results. As results of the aforementioned study were promising, more than 100 case reports have since been published with up to 90 percent of the cases treated with FMT showing successful results.^40^

The prevalence of CDI is of 7.9% in Qatar, with 5.9 cases per 1000 pediatric patients and 1.6 cases per 10,000 adult patients.

Six clinical trials (using mixed methods and sample sizes) are listed on *clinicaltrial.gov* and have published their results; these are compiled in Table 1. Overall, results described a very good clinical resolution rate for diarrhea of about 70%–100% with minimal adverse events.

#### Inflammatory bowel diseases

**Figure 5.**
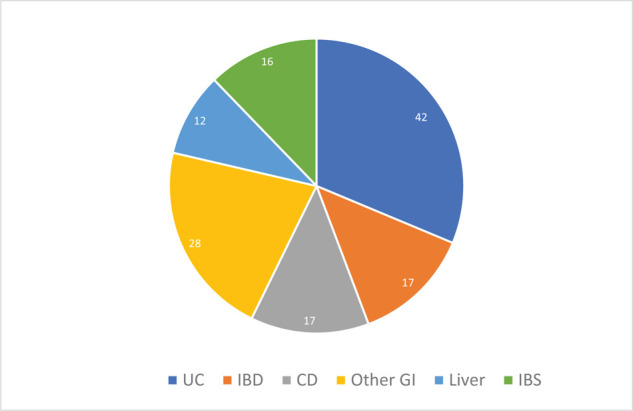
**Proportions of clinical trials for GI diseases and disorders**. Abbreviations: UC = ulcerative colitis; IBD = inflammatory bowel disease (nonspecified); CD = Crohn's disease; Other GI = other gastrointestinal disorders; Liver = liver diseases; IBS = irritable bowel syndrome. Data were obtained from clinicaltrials.gov as per method detailed in section 2.

Another application of FMT is the treatment of GI diseases and disorders and more specifically IBD. Eleven studies registered on *clinicaltrials.gov* have published their results so far. Crohn's disease and ulcerative colitis are inflammatory bowel diseases for which prevalence is increasing. These IBDs are also found as comorbidity factors in other diseases and disorders such as ASDs. Seventy-five clinical trials on IBD and FMT are active and registered on the database (Figure 5).

Mixed results have been reported ranging from no clinical remission to full remission and maintenance of remission of the disease. The trials described in Table 2 highlight important key principles relating to the use of FMT in IBD. Importantly, the gut microbial diversity is reestablished by the FMT; very often the microbial diversity of the acceptor becomes similar to the microbial diversity of the donor. However, the clinical resolution of symptoms depends on various factors including the following:

1. The number of FMTs performed and the time elapsed between them.
2. The severity of the disease: some refractory ulcerative colitis did not see any improvement after single FMT. Further trials are needed to find out if increasing the number of FMT or their regularity would be beneficial.
3. The donor's microbiome: some statistical significance have been found when comparing donors suggesting a donor dependence.^41^

These trials reveal that various protocols have been developed and tested. Examples include the use of single FMT versus multiple FMT over a period of time; mode of delivery (colonoscopy, nasojejunal tube, gastroscopic infusion, or self-administration of enema), administration with and without prior use of antibiotics, or other premedication.

Recent meta-analyses concluded that FMT is relatively safe and significantly more successful than placebo in treating ulcerative colitis.^42^ FMT showed it could constitute an effective treatment with less adverse events when compared to biological agents that act against the immune system.^43^

#### Diabetes

Type 2 diabetes (T2D) is a common metabolic disorder characterized by decreased insulin sensitivity and impaired islet cell function; it can be caused by genetic and environmental factors. Dysbiosis may induce chronic inflammation of pancreatic islets. This may lead to damage of β-cells causing apoptosis, which is known to be associated with type 2 diabetes.^44^ A recent study showed promising results of the use of FMT in tbl2D in mice models. FMT was administered over a period of eight weeks to mice who had been fed a high-fat diet combined with streptozotocin for six weeks prior to the treatment.^44^ The results indicated a number of improvements in symptoms associated with diabetes. Insulin sensitivity increased and damage to the islets was significantly reversed after the treatment; this was accompanied by a reduction of the inflammatory response. This result suggests promise for reversing tbl2D in humans. Two human clinical trials are currently testing various protocols of FMT to treat tbl2D. However, none have published results at this time.

#### Obesity

The role of dysbiosis in obesity may include changes in molecular signaling molecules released by bacteria and the translocation of intestinal bacteria and their products; as a result, paracrine and other endocrine signals are affected. Gut microbiota communicates with the host's adipose tissue, liver, and brain. One of the proposed mechanisms linking dysbiosis to obesity is that secretion of hormones by enteroendocrine cells could be modified by several species of gut bacteria, resulting in reduced satiety.^45^

Many animal studies have been performed worldwide that use the germ-free mice model over the past few years; some of the results suggest that FMT may provide a potential treatment to reduce obesity. Germ-free mice inoculated with microbiota from obese or lean human twins took on the microbiota characteristics and the obese phenotype of their donor. Other studies showed that FMT reverses obesity in mice.^23^

Twelve studies were reported after excluding those that were suspended, terminated, or withdrawn. However, only one of them had published results specific to obesity. This randomized, triple-blinded, placebo-controlled trial used an induction dose of FMT via capsules, followed by monthly oral capsules or placebo capsule for maintenance. FMT capsules did not cause any adverse event and led to significant changes in the gut microbiome; bile acid profiles of participants shifted to become similar to those of the lean donor. However, FMT did not reduce body mass index (BMI) in obese, metabolically uncompromized patients. Although the preclinical studies seem very promising, further clinical studies, using different protocols, are still needed to support the use of FMT in the treatment of obesity.

#### Autism spectrum disorders

ASDs are a spectrum of neurobiological disorders that impair communication skills and social interaction and lead to repetitive, restricted behaviors and activities. As ASDs have been associated with significant dysbiosis and microbial diversity shifts, a growing interest for the modulation of the gut microbiome in ASD is being observed worldwide. Dysbiosis is associated with the increased intestinal permeability of exogenous dietary molecules, inflammatory cytokines, and bacterial products such as lipopolysaccharide (LPS), which are known to be neurotoxic. Therefore, dysbiosis may link the gut microbiota and the related metabolites to what is called the “gut–brain axis.”^46^ Some preclinical studies have shown very promising results for the reversal of autism-like symptoms in mice models using probiotics or fecal transplants; four clinical studies listed on the database are exploring the effectiveness of fecal transplants on ASD. One of them, perhaps the only one of its scale for neurodevelopmental disorders, was conducted at Arizona State University and has recently published its results. In this study, a modified FMT treatment plan was formed with multiple administrations of donor microbiota to 18 pediatric patients with ASD and GI problems. Patients underwent 14 days of oral vancomycin treatment followed by a bowel cleansing and a flourishing of the gut microbiota through FMT either orally or rectally. Patients were monitored for eight weeks and again two years after this treatment ended to determine if the effects of the treatment were long-lasting.^47^ The number of children with severe symptoms decreased by 50% after eight weeks and to approximately 80% after two years. Both the gastrointestinal and ASD symptoms were resolved. Abdominal pain, indigestion, diarrhea, and constipation showed substantial improvement. ASD symptoms related to speech, communication, and social skills also showed remarkable improvement. The gut microbiota of ASD individuals reflected the positive changes with an increase of certain beneficial bacteria that were previously reported as underrepresented in children with ASD. Up to a fourfold increase in the abundance of *Bifidobacterium*, *Prevotella*, and *Desulfovibrio* was noted, and the microbiome became comparable in abundance to that of neurotypical children. These changes persisted weeks after the treatment, which could be interpreted as an effective FMT protocol. This important, long-lasting feature of the treatment might, in reality, be related to the fact that all of the individuals in this study were children; this suggests that the nonmaturity of the gut microbiome at younger age might be a good prognostic factor for the long-term success of the intervention. Similarly, for other diseases data suggest that earlier interventions in relation to the diagnosis of the disease seem to have better rate of success (Table 2).^41^

## Discussion

Fecal microbiota transplants aim to address a new and emerging theory in medical literature—the importance of the human gut microbiome and its central role in understanding how disease can be understood and treated. Many diseases have been associated specifically with dysbiosis or decreased bacterial diversity of gut microbiota—the bacteria, phages, archaea, fungi, viruses, and the billions of microorganisms present in the human body.^48^ It was initially thought that gut microbes only served to control the population of pathogenic bacteria in the body. However, now it is known that the microbiota plays a vital role in the production of essential metabolites and, more importantly, the regulation of the immune system.^48^ Changes in environment, diet, and even genetics have corresponded to abrupt and even chaotic changes in the gut.

The composition of the gut microbiota is a key factor in understanding the pathogenesis and prognosis of various diseases, including infectious diseases such as CDI. FMT has been shown to result in clinical resolution in 80%–90% of patients with CDI. A balanced microbiota was shown to be the main protective barrier against CDI as confirmed by meta-analysis.^49^ Some of the mechanisms include the secondary bile acids produced by gut bacteria that inhibit the germination of *C. difficile* spores.^50,51^ When eubiosis is disrupted, *C. difficile*, which is a normal member of the microbiota, starts to dominate and colonize the large intestine. This colonization may lead to the disruption of epithelial barrier integrity resulting in the leaking of toxins and inflammation.^52^ The mortality rates related to *C.* *difficile* infections in United States increased between 1999 and 2004 from 5.7 per million population to 23.7 per million,^37^ before stabilizing at this level with the introduction of FMT for the recurrent CDI infections.

There has been a surge in clinical trials and studies of FMT to determine its effectiveness for a spectrum of diseases after its success. Recent studies have evaluated the long-term impacts of fecal transplants on the gut microbial composition. The microbial shift toward the donor's gut microbes has been shown to persist after one year in healthy individuals^53^ and two years in patients with *C.* *difficile* infection.^54^ An increased gut biodiversity has been shown after two years in a study conducted on ASD.^55^ For this reason, some believe in a microbiome “fingerprint” that could hold the key to increased understanding of the dynamics of health and disease. Xia et al. (2019) developed the “Stroke Dysbiosis Index” and reported a strong association between dysbiosis and stroke outcome in a mouse model.^56^ On May 28, 2020, the first FMT trial sponsored by the Chinese Academy of Medical Sciences, Fuwai Hospital was registered on *clinicaltrial.gov* database for the treatment of hypertension using oral capsules (NCT04406129).

Currently, more than 200 clinical trials on FMT are listed on the database *clinicaltrials.gov*, many of which are still ongoing. This indicates that with more human clinical trials the understanding of FMT and its applications for other diseases beyond CDI might expand to include more trials for neurodegenerative (Alzheimer's disease, Parkinson's disease), neuropsychiatric (schizophrenia, anxiety, depression), neurodevelopmental (ASDs, attention deficit hyperactivity disorders) inflammatory bowel diseases (Crohn's disease, ulcerative colitis), celiac disease, dermatitis, asthma, liver diseases, cancer, diabetes, obesity, and cardiovascular diseases.^57^ A recent randomized controlled trial run in Qatar and published by Taheri and colleagues (2020) reported that an intensive lifestyle change can reverse diabetes^58^; analysis of the gut microbiome and metabolome evolution during such lifestyle changes could lead to the consideration of FMT for acute treatment of dysbiosis.^59,60^

### Challenges in evaluating effectiveness of FMT

The vast majority of information related to the effectiveness of FMT was derived from case studies; this limited the accuracy of assessments and comparisons to standard techniques and treatments. However, the understanding of efficacy of FMT is expected to improve with the growing number of controlled randomized and blind clinical trials.

Several variables can influence the effectiveness of FMT and need to be controlled before drawing any conclusions regarding its effectiveness for treating specific diseases or disorders:

1. Donor's fecal sample characteristics: Fecal samples might need to be classified based on the microbiome and metabolome profiles in addition to screening for pathogens and antibiotic resistance to develop a more personalized FMT.
2. Patient's characteristics: Severity of the disease might impact the efficacy of FMT, and personalized protocols might be needed. Lack of awareness of the potential of FMT renders the recruitment of participants to trials difficult. This also reduces the chances of creating trials that mainly test the efficacy of FMT on disease severity. A study by McLeod and colleagues (2019) analyzed the social representations of feces and gut microbes by investigating English language media coverage of the topic. This study results showed that the media progressively contributes to a change in public opinion toward feces and gut microbes.^61^
3. Patient's preparation: Some preparations were shown to trigger inflammation. For example, polyethylene glycol (PEG) is sometimes used for bowel cleansing to dislodge the endogenous intestinal microbiota prior to FMT. This type of preparation could eventually trigger new flares in patients with IBD and impair further assessment of the FMT. No trials have been made to date to compare the impact of patient's preparation on effectiveness of FMT via colonoscopy.
4. Method of delivery: The biggest challenge is to establish protocols for groups of conditions based on variables that can be assumed to influence the efficacy of the FMT. The variables collected in this study were related to (1) sample consistency: whole, fresh, frozen, filtered, and lyophilized; (2) the mode of delivery: enema, capsule, and nasogastric; (3) the number of FMT courses: single or multiple; (4) the addition of antibiotics or not; (5) the addition of other drugs or not. All these variables are adding a further challenge to the evaluation of effectiveness of FMT as a new therapeutic approach. A recent meta-analysis by Ramai and colleagues (2020) has investigated the different modes of delivery of FMT and concluded that colonoscopy was more efficient than the use of enema or nasogastric tube in the case of *C.difficile* treatment. Interestingly, FMT performed with capsules were found to be of similar efficiency to FMT using colonoscopy.^62^ Nevertheless, further studies are needed to better define optimal methodology of the FMT delivery as confirmed by a meta-analysis.^63,64^
5. Measurement of outcomes: Most trials measure clinical resolution and long-term efficacy by following up with patients for eventual relapse. Despite being an important outcome measurement for infectious diseases such as the recurrent CID, it is necessary to highlight the fact that FMT has no effect on the factors that originally cause dysbiosis in the case of noncommunicable diseases (NCDs), most of which are related to lifestyle habits. Therefore, FMT is no more than an acute change of the gut microbiome that can only solve dysbiosis and its resulting health problems in the case of NCDs. The long-term effectiveness of FMT based on relapses in the cases beyond CDI or infectious diseases is not a scientifically sound approach. Sokol and colleagues (2020) reported that the patient's alpha diversity returned to the original diversity after 14 weeks of FMT.^27^ However, this study does not necessarily indicate the inefficiency of FMT. It should not be expected that FMT can cure a noncommunicable disease when the root causes have not been addressed. If dysbiosis is caused by the lifestyle habits pursued by the patient, there is a high probability that a relapse will occur. However, if the causes of dysbiosis are addressed simultaneously, or if the gut microbiota maturation is still at its early stages, such as in children, FMT would be the best therapeutic approach that “resets” the gut to a normal state. The outcomes measured should include checking the alpha diversity and species richness of fecal samples of patients at follow-up as has been done in some studies.^41^

### Risks of FMT

While FMTs seem to hold promise for many diseases and their positive effects speak volumes based on the emerging data, recent events have triggered a greater need to reevaluate the donor stool screening measures and address the potential harms of growing antibiotic resistance. An important risk directly related to FMT has emerged in addition to the risks inherent in the methods of delivery itself (such as the risk of mucosal tear during colonoscopy).^65^ Two patients died in 2019 because of multi-drug resistance acquired through FMT.^66^ The Federal Drug Administration responded with stricter guidelines^67^ to screen for antibiotic-resistant bacteria in fecal samples. This incident highlighted a deeper, rapidly growing issue that was directly and indirectly affecting humans and the environment. The growth of antibiotic resistance was posing a threat to several medical practices despite the medical advances gained with the discovery of antibiotics. This has the potential to compromise many medical treatments and is further exacerbated by the lack of treatment for pathogens that are resistant to antibiotics. The overuse and misuse of antibiotics for both humans and agricultural life have produced an increased number of antibiotic-resistant pathogens; bacterial pathogens containing resistant genes are constantly released into ecosystems, and the extent of their effects on humans and the environment is still unclear.^68^ From medicine to food to the environment, it could be argued that many individuals may have developed some form of antibiotic-resistant microbiota because of multiple prolonged exposures to antibiotics. In light of this FMT as a potential effective acute treatment of dysbiosis and its consequences could be hindered as antibiotic resistance is becoming a criterion of selection of donor stool samples. Antibiotic resistance could wipe out the new hope that FMT is raising to treat many diseases and disorders.

Moreover, some deaths following FMT have been attributed to other causes such as heart attack^69^; this raises the question of screening the samples for metabolites. Studies on the gut–heart axis have acknowledged the important role played by trimethylamine (produced by gut bacteria) in increasing the amount of trimethylamine oxide, a metabolite that was shown to be involved in cardiovascular diseases.^57^ Studies on FMT must consider the metabolomic and genomic studies that investigate the association between microbiome prints and diseases; an understanding of the mechanisms by which certain metabolites found in feces can act systemically, (including crossing the blood–brain barrier) is essential.

FMT could become a very successful approach in the treatment and management of diseases but only after some advances are made. Such advances include composition of the fecal samples, method of administration, and consideration of moving from simple screening to a real personalization of the fecal sample used. McGovern and colleagues (2020) in their study ran a gap analysis and proposed some recommendations to manage possible risks in the design of future trials that could involve FMT.^70^

## Conclusion

This review reports latest developments on FMT and its current clinical applications by giving an overview of the clinical trials registered in *clinicaltrial.gov* database. While its primary application is the *C.* *difficile* infection, FMTs are being tested on a large panel of diseases and disorders including gastrointestinal diseases, cancers, neurological diseases, neurodevelopmental disorders, antimicrobial resistance, and many more.

Results of trials for CDI and IBD constituted approximately 50% of the FMT trials registered on the database. Trials reported it was an optimal approach for CDI and promising one for IBD in using FMT as a treatment, and these are confirmed by several meta-analyses.

Several meta-analyses have now concluded on the superiority of FMT compared to placebo in treating *C.* *difficile* infections and forms of IBD, leading to clinical resolution, remission, or even cure of the infection. As prevalence of CDI in Qatar and atypical cases of IBD are calling to local surveillance, this review is a call to consider this rediscovered approach for research and treatment of these diseases in Qatar.

This study also reported extradigestive applications of FMT which are of a particular interest in Qatar because of their relatively high prevalence as compared to the world average and for being national health priorities. Results were scarce for diabetes and obesity and mixed in regard to improvements; further studies are needed to better define optimal methodology of the FMT delivery. Studies showed promising results, long-term benefits of the FMT with respect to ASD including microbial shifts toward the donor's microbiome with an increase of *Bifidobacterium* and *Prevotella* to, respectively, 5- and 82-folds after 2 years and a reduction from severe to mild or no autism in a significant proportion of the cohort. FMT is proposed as a novel approach to investigate on autism in Qatar.

The study highlighted the potential risks of FMT and the necessity of monitoring transfer of antibiotic resistance, which is the main significant risk related to FMT. Variations of the FMT method and other factors such as sample origins have been shown to contribute to the heterogeneity of results published on the effectiveness of FMT, and this should be taken into consideration when designing future studies.

The growing prevalence of NCDs creates a need for more treatment options. With the rapid advances of metabolomic and microbial prints of diseases, growing evidence is advocating for a role of the gut dysbiosis in the pathogenesis of chronic diseases and disorders. Fecal transplants could become an interesting approach to “reset” the gut to microbial homeostasis and minimum inflammatory status. This review is a call to perform preclinical studies on animal models to test the effectiveness of fecal transplants in managing chronic diseases in Qatar.

In summary, this review features the potential benefits and risks of FMT and the necessity to invest in such preclinical and clinical trials in the Middle East.

### Competing interests

None of the authors have competing interests to declare.

### Author's contributions

DA and AA contributed to data acquisition, data analysis and interpretation and revision of the manuscript. AS and DZ contributed to conception, writing and revisions. CM and AC contributed to design, writing and revisions and GB has conceived, designed, drafted and revised the manuscript. All authors read and approved the final manuscript.

### Funding

This work was supported by the Qatar National Research Fund. Grant Number: 5727002821.

## Figures and Tables

**Table 1:** Clinical trials on CDI that have published their results: methodology and clinical resolutions.

Reference	Condition	Method of delivery	Participants	Results

Youngster et al.^71^ (2014)	rCDI	Discontinuation of antibiotics 48 hours prior to FMT + fasting 4 hours + 15 capsules intake of 1.6 g/capsule from single donor + fasting 1 hour for 2 consecutive days.	Twenty participants with at least 3 episodes of mild to moderate *C. difficile* infection + failure of a 6- to 8-week vancomycin taper or at least 2 episodes of severe *C. difficile* infection requiring hospitalization were enrolled	Resolution of diarrhea was achieved in 14 patients (70%; 95% CI, 47%–85%) after one course of FMT. Four patients did not respond at first then had resolution of diarrhea after retreatment. Overall clinical resolution rate was 90% (95% CI, 68%–98%)

Kelly et al.^65^ (2014)	Immunocompromised patients with recurrent, refractory, or severe CDI.	Methods of delivery were varied. Retrospective study of patients with CDI who underwent FMT at 16 medical centers nationally and internationally. Mean follow-up period between FMT and data collection was 11 months (range 3–46 months).	Adults (75) and children (5) treated with FMT for recurrent (55%), refractory (11%), and severe and/or overlap of recurrent/refractory and severe CDI (34%)	Clinical resolution after single FMT was 78%. Twelve patients underwent repeat FMT followed by clinical resolution. Overall clinical resolution was 89%. Sixty-two patients had no recurrence at least 12 weeks post-FMT.

Lee et al.^72^ (2016)	*C. difficile* infection recurrent or refractory	Suppressive antibiotics for most recent episode of CDI + discontinuation of antibiotic 24–48 hours prior to FMT + delivery of frozen or fresh 50 mL enema. Repeat FMT from same donor if no resolution at day 4. Enema preparation: 100 g of stool diluted in 300 mL of water, and filtration of solids using gauze. Suspension is used within 24 hours of thawing.	Population 1: 219 patients (n = 108 in the frozen FMT and n = 111 in the fresh FMT) Population 2: 178 (frozen FMT: n = 91, fresh FMT: n = 87)	Population 1: The clinical resolution was 75% for the frozen FMT group and 70.3% for the fresh FMT group. Population 2: Clinical resolution was 83.5% for the frozen FMT group and 85.1% for the fresh FMT group.

Hvas et al.^73^ (2019)	rCDI	Use of vancomycin (125 mg 4 times daily) for 4–10 days or 10 days of fidaxomicin (200 mg twice daily) followed by FMT by colonoscopy or nasojejunal tube.	All 64 patients received their assigned treatment	Clinical resolution was observed in 92% of patients given FMTv (24 patients), 42% given fidaxomicin (24 patients), and 19% given vancomycin (16 patients).

Ianiro et al.^69^ (2018)	rCDI	Two treatment arms: FMT-S: A 3-day pretreatment with vancomycin (250 mg/4 times a day), 2 L of macrogol bowel cleaning followed by a single fecal infusion by colonoscopy and 14-day vancomycin course (250 mg/four times a day). FMT-M: A 3-day pretreatment with vancomycin (250 mg/four times per day), 2 L of macrogol bowel cleaning followed by at least two fecal infusions by colonoscopy every 3 days and a 14-day vancomycin course (250 mg/4 times a day). Samples: At least 50 g of fresh or frozen feces.	Fifty-six patients, 28 in each treatment arm	Clinical resolution: FMT-S: 75% FMT-M: 100% A transient clinical improvement was observed immediately after FMT in all of them, but many of them experienced a relapse of diarrhea and worsening of their clinical picture 10 days after treatment.

Camacho-Ortiz et al.^74^ (2017)	CDI	Two arms were tested: Standard: Oral vancomycin (250 mg every 6 hours for 1014 days) FMT-FURM: Fecal donorunrelated donor pooling of samples from multiple donors; 3 samples/donor collected every 2 weeks. Resuspended in 0.9% saline and filtered to remove particles>330 μm. Glycerol, at a final concentration of 15% (v/v), as a bacterial cryoprotectant before storage. Each dose was 45 mL aliquot.	Standard arm: 9 patients FMT-FURM arm: 7 patients	Clinical resolution: Standard arm: 88.9% FMT-FURM arm: 57.1% after the first dose, 71.4% after the second dose.


**Table 2:** Clinical trials on IBD which have published their results: methodology, clinical resolutions, and remissions.

Study title/reference	Condition	Method	Participants	Results (efficacy, failures/deaths)

Paramsothy et al.^75^ (2017)	Active UC	Bowel preparation + colonoscopy with 150 mL of either donor or placebo. Colonoscopy after bowel preparation to administer initial infusion + self-administration of enemas 5 times per week (5 days on and 2 days off) for 8 weeks for a total of 40 enemas.	FMT: 41 patients Placebo: 40 patients	Clinical resolution at 8 weeks: 44% for FMT. Endoscopic response: 32% for FMT versus 10% for placebo.

Jaboc et al.^76^ (2017)	Active UC	Single FMT delivery by colonoscopy with a two-donor fecal microbiota preparation.	Twenty patients.	Clinical resolution at 4 weeks: 35% Remission at 4 weeks: 15%, 10% with mucosal healing. Needed escalation: 15%

Ding et al.^77^ (2019)	Moderate-to-severe UC	Step-up FMT included: Step 1: Single fecal microbiota transplantation Step 2: If no clinical resolution, two or more FMT over several days. Step 3: If no clinical improvement, FMT + immunosuppressants/corticosteroids If patients achieved clinical improvement after FMT, they would be recommended to administer the second course of FMT 3 months later to maintain the clinical benefits from the first course.	One hundred and nine patients	Clinical response at: 1 week: 73.4% 1 month: 74.3% 3 months: 51.4% 6 months: 28.4% Remission achieved at: 1 week: 30.3% 1 month: 25.7% 3 months: 20.2% 6 months: 13.8%

Sokol et al.^78^ (2020)	Colonic or ileocolonic CD	Clinical remission using corticosteroids followed by after colon cleansing (4 L of polyethylene glycol) + single fecal transplant of 50–100 g of filtered stool from a donor resuspended in 250–350 mL of sterile sodium chloride.	Sham: 10 patients FMT: 11 patients	Alpha diversity: Significant increase in alpha diversity following FMT but not sham. However, this change returned to its initial level 14 weeks after FMT.

Kump et al.^79^ (2013)	Chronic active UC refractory to conventional medical therapy	Single FMT: Bowel lavage using a standard PEG solution + colonoscopy to infuse fractions of 20 mL of the donor’s fecal solution into the ileum and colon until a total of 300–500 mL was transferred followed by received 4 mg of loperamide to slow intestinal transit.	Six patients	Improvement at 2 weeks: 100% Remission at 90 days: 0% FMT was associated with a temporal increase in species richness in fecal and mucosal samples of the patients (not significant).

Moayyedi et al.^41^ (2015)	Active UC	Single FMT: 50 mL via enema from healthy anonymous donors or placebo 50 mL water enema once weekly for 6 weeks.	FMT: 36 patients Placebo: 34 patients	Remission at 7 weeks: FMT: 24% Placebo: 5% Biopsies of the patients in remission at 7 weeks: FMT: No active inflammation in any biopsy Placebo: 2 patients had mild patchy inflammation in the rectum with no active inflammation in sigmoid and descending colon biopsies. Microbial diversity: FMT patients had greater microbial diversity compared with baseline than that of patients given the placebo. Other findings: Treatment successes attributable to donor B were 39% versus 10% with other donors (*p* = 0.06, Fisher's exact test), suggesting statistical evidence for donor dependence. Trend toward those taking immunosuppressant therapy to have a greater benefit from FMT 46% versus 15%. Patients with a recent (less than a year) diagnosis of UC were statistically significantly more likely to respond to FMT (75% vs 18%; *p* = 0.04, Fisher's exact test).

Rossen et al.^80^ (2015)	Mild to moderately active UC	Two arms: FMT: Feces from healthy donor. Control: Autologous FMT. Lavage + fresh FMT via nasojejunal tube with 500 mL fecal suspension. Repeated at 3 weeks.	FMT: 23 patients Control = 25 patients	Clinical remission (simple clinical colitis activity index scores ≤ 2): FMT: 30.4% Control: 20%

Wang et al.^81^ (2018)	Mild-to-severe CD	Single FMT: Purified FMT via nasojejunal tube or gastroscopic infusion. Second FMT at 13 months or 36 months.	Per follow-up time: 12 months: 139 patients 2 years: 106 patients 5 years: 32 patients	Clinical response rate and clinical remission rates were, respectively, of 45% and 20% in the patients with adverse events, which was significantly lower than 75.6% and 63% in patients with no adverse events.

He et al.^82^ (2017)	CD complicated with intra-abdominal inflammatory mass	The one-hour FMT protocol. Initial FMT followed by repeated FMTs every 3 months.	Twenty-five patients	Clinical response and clinical remission were respectively of 68% and 52% at 3 months post the initial FMT. Sustained remission was at 6 months 48%, at 12 months 32%, at 18 months 22.7%.

Suskind et al.^83^ (2015)	Pediatric Crohn’s disease	Rifaximin 200 mg 3 times daily for 3 days until the evening before procedure + omeprazole (1 mg/kg orally) on the day before and morning of the procedure + 1 capful of MiraLAX in 8 oz of water 3 times a day for 2 days + FMT of 30 g of donor stool was mixed with 100–200 mL of normal saline gauze filtered administered by nasogastric tube.	Nine patients	The mean pediatric Crohn’s disease activity index score improved with patients having a baseline of 19.7 ± 7.2, with improvement at 2 weeks to 6.4 ± 6.6, and at 6 weeks to 8.6 ± 4.9. Remission at 2 weeks: 7/9 patients Remission at 6 and 12 weeks: 5/9 patients (did not receive additional medical therapy).

